# Bronchiectasis in Inborn Errors of Immunity: Prevalence, Predictors, and Cardiopulmonary Complications in a Genetically Characterized Cohort

**DOI:** 10.1007/s10875-026-02044-8

**Published:** 2026-07-08

**Authors:** Diana Marangu-Boore, Katherine Myint-Hpu, Esther Kang, Luigi D. Notarangelo, Ottavia M. Delmonte

**Affiliations:** 1 Laboratory of Clinical Immunology and Microbiology, National Institute of Allergy and Infectious Diseases, National Institutes of Health, Bethesda, MD USA; 2 Pulmonology Division, Department of Paediatrics and Child Health, University of Nairobi, Nairobi, Kenya

**Keywords:** Adults, Bronchiectasis, Cardiopulmonary Outcomes, Children, Chronic Lung Disease, Hematopoietic Stem Cell Transplantation, Primary Immunodeficiency Disorders, Pulmonary Hypertension

## Abstract

**Purpose:**

Bronchiectasis poses a serious but incompletely defined burden in patients with inborn errors of immunity (IEI). We determined its prevalence, independent predictors, and cardiopulmonary complications in a genetically characterized IEI cohort to inform care in this vulnerable population.

**Methods:**

We conducted a cross-sectional analysis of patients enrolled in a National Institutes of Health prospective IEI protocol (2018–2024) who had undergone whole exome or genome sequencing and chest computed tomography (CT). Bronchiectasis was radiologically confirmed, excluding traction bronchiectasis. Predictors were identified by multivariable logistic regression. Cardiopulmonary outcomes included spirometry, lung volumes, diffusing capacity, six-minute walk distance, pulmonary hypertension by echocardiography, and mortality.

**Results:**

Of 229 enrolled patients, 131 were eligible, representing 31 distinct IEIs. Median age at first chest CT was 20 years (IQR 10–33). The most common CT finding was pulmonary nodules (55%). Bronchiectasis prevalence was 27% (95% CI 20–35%). Independent predictors were older age at first CT (aOR 1.04, 95% CI 1.01–1.08), combined immunodeficiency affecting cellular and humoral immunity (aOR 4.30, 95% CI 1.42–14.54), predominantly antibody deficiencies (aOR 5.83, 95% CI 1.66–22.26), and diseases of immune dysregulation (aOR 7.56, 95% CI 1.07–52.72). Patients with bronchiectasis had greater cardiopulmonary impairment across all measured domains and higher mortality (15% vs. 3%; *P* = 0.046).

**Conclusions:**

Bronchiectasis is common across IEI diagnostic classes and carries substantial cardiopulmonary morbidity and excess mortality. Older age at first chest CT and specific IUIS classes, particularly predominantly antibody deficiencies and diseases of immune dysregulation, independently predict bronchiectasis, identifying patients who would benefit most from early pulmonary surveillance.

**Trial Protocol:**

NCT03394053.

## Introduction

Bronchiectasis is a heterogeneous chronic pulmonary condition associated with significant morbidity in children and adults [1]. Patients with inborn errors of immunity (IEI) are highly susceptible to bronchiectasis due to recurrent infections, impaired epithelial and mucociliary function, chronic inflammation and progressive structural lung damage, four interrelated factors that constitute the “vicious vortex” of disease progression [2, 3]. The 2024 International Union of Immunological Societies (IUIS) Expert Committee report recognizes 555 IEIs and 17 phenocopies linked to approximately 500 different genes, with over 250 novel immune genes identified in the last decade [4, 5]. Beyond genetic predisposition, patients with IEI may have additional risk factors for bronchiectasis including smoking, indoor air pollution, and undernutrition, all fundamental to the genome-exposome paradigm [3, 6].

Uniquely, susceptibility to bronchiectasis in IEI may be identifiable at birth through genomic based newborn screening [7, 8], or early on in life through immunological phenotyping and genetic testing, offering a critical window to prevent, halt, delay or reverse bronchiectasis progression. The IUIS classification organizes IEIs into ten diagnostic groups, and highlights conditions associated with respiratory phenotypes [4, 9]. Although recurrent pneumonia is a prominent feature across several diagnostic groups, only 12 conditions in the current classification are explicitly linked to bronchiectasis, likely an underestimate [4]. Importantly, a diagnosis of bronchiectasis may directly influence curative IEI treatment decisions, accelerating the timeline to intervention, modifying the treatment strategy, or in some cases, serving as a contraindication.

Data on the prevalence, predictors, and cardiopulmonary consequences of bronchiectasis across the full spectrum of IEI remain scarce, with no large genetically characterized cohort study to date comprehensively addressing all three. Understanding these outcomes is essential to guide surveillance strategies, optimize management, and inform curative treatment decisions in this rare and vulnerable population.

## Methods

Protocol NCT03394053 (“The Mechanistic Biology of Primary Immunodeficiency Disorders”) is an ongoing prospective cohort study approved by the National Institutes of Health (NIH) Institutional Review Board, aimed at understanding the natural history and underlying mechanisms of IEI. All study participants, or their parents provided written informed consent and age-appropriate assent according to the Declaration of Helsinki [10]. Patients aged two years and older with a confirmed or suspected IEI were eligible for enrollment. Clinically indicated and research investigations included full blood counts, immunoglobulin levels, detailed lymphocyte subsets, and genetic testing by whole exome or whole genome sequencing. Additional investigations, including computed tomography (CT) of the brain, sinuses, chest, abdomen, and/or pelvis, were ordered based on each patient’s clinical phenotype. Study data were entered prospectively into secure online clinical and research databases. Investigations performed at external sites were uploaded to these databases and CT images were reviewed by the research center’s radiologists.

### Study Design

We conducted a cross-sectional analysis among children and adults enrolled in the NCT03394053 protocol between 2018 and 2024. Eligible patients were required to have undergone chest CT, which served as the primary inclusion criterion for this analysis. The objectives were to determine the prevalence, predictors, and complications of bronchiectasis in this cohort, and to examine whether the presence of bronchiectasis influenced curative IEI treatment decisions.

### Predictors

All predictors were identified a priori. Demographic characteristics including date of birth, sex and race were captured at enrollment. Specific genetic variants were identified through Sanger, whole exome or whole genome sequencing performed by the institute’s genetic testing core program according to protocol NCT03206099 (National Institute of Allergy and Infectious Diseases Centralized Sequencing Protocol) approved by the NIH Institutional Research Board following previously published methodology [11, 12], and/or based on prior genetic reports.

We used the IUIS expert committee’s 2024 update on the classification of human IEI to classify study participants based on laboratory phenotype and/or genetic testing results [4, 9]. Patients were designated as unclassified when a molecular diagnosis was not established and the laboratory phenotype did not permit unambiguous assignment to a single IUIS diagnostic class.

### Outcomes

All patients in this analysis had chest CT. Bronchiectasis was ascertained on chest CT as reported by any of the clinical research center’s radiologists. Traction bronchiectasis denotes irreversible bronchial dilatation and distortion secondary to mechanical traction from adjacent fibrotic lung, and on chest CT it classically localizes to areas of peripheral or subpleural fibrosis with accompanying reticulation or honeycombing [13]. For this analysis, bronchiectasis was defined to exclude traction bronchiectasis, a manifestation of interstitial lung disease (ILD), so as to focus on airway-wall destruction attributable to infection or inflammation, consistent with established non-CF bronchiectasis guidelines [1, 14].

Granulomatous-lymphocytic interstitial lung disease (GLILD) is a non-infectious, immune-mediated ILD frequently associated with common variable immunodeficiency and other primary immunodeficiencies, characterised histologically by pulmonary non-necrotising granulomas and/or lymphoid proliferation. On chest CT, GLILD is suggested by a constellation of findings including multifocal pulmonary nodules, patchy or diffuse ground-glass opacities often with peribronchovascular or airway-centred distribution, and mediastinal or hilar lymphadenopathy [15]. For the purposes of this study, GLILD was defined radiologically by the concurrent presence of pulmonary nodules, ground-glass opacities and mediastinal or hilar lymphadenopathy on chest CT.

Cardiopulmonary complications were measured by lung function testing, six-minute-walk testing and transthoracic echocardiography (TTE). Lung function testing comprised spirometry; lung volumes measured by body plethysmography or nitrogen washout; and diffusion capacity of the lungs for carbon monoxide (DLCO). We applied global race-neutral equations and the European Respiratory Society (ERS)/American Thoracic Society (ATS) technical standard approach to interpreting lung function tests [16, 17]. Six-minute-walk distance in children and adults was classified as abnormal based on published reference equations [18–20]. We utilized peak tricuspid regurgitation velocity > 2.8 m/s or tricuspid annular plane systolic excursion < 18 mm assessed via TTE as non-invasive parameters of pulmonary hypertension [21].

For the purposes of this analysis, the terms “first” and “last” refer to the earliest and latest imaging or investigations available within the study time period (2018–2024), respectively, and do not necessarily reflect the first or last in the patient’s lifetime.

### Bronchiectasis Diagnosis Influencing Curative Treatment Decision

We also assessed whether a diagnosis of bronchiectasis influenced decisions regarding definitive curative treatment. Curative options for IEIs, such as hematopoietic stem cell transplantation (HSCT), gene therapy, or thymus implantation, are guided by the specific underlying genetic defect.

### Statistical Methods

Patients’ descriptive characteristics were summarized as frequencies and percentages for categorical variables and medians and interquartile ranges (IQR) for continuous variables. We estimated the prevalence of bronchiectasis among patients with IEI and provided a 95% confidence interval (CI). Additionally, we modelled predictors of bronchiectasis using multivariable logistic regression. Age, sex, race and IUIS class were predictors we considered a priori for our multivariable model. Statistical analyses were performed using R version 4.3.3. Values of *P* < 0.05 were considered statistically significant and all *P*-values were two-tailed.

## Results

### Patient Characteristics

**Fig. 1:**
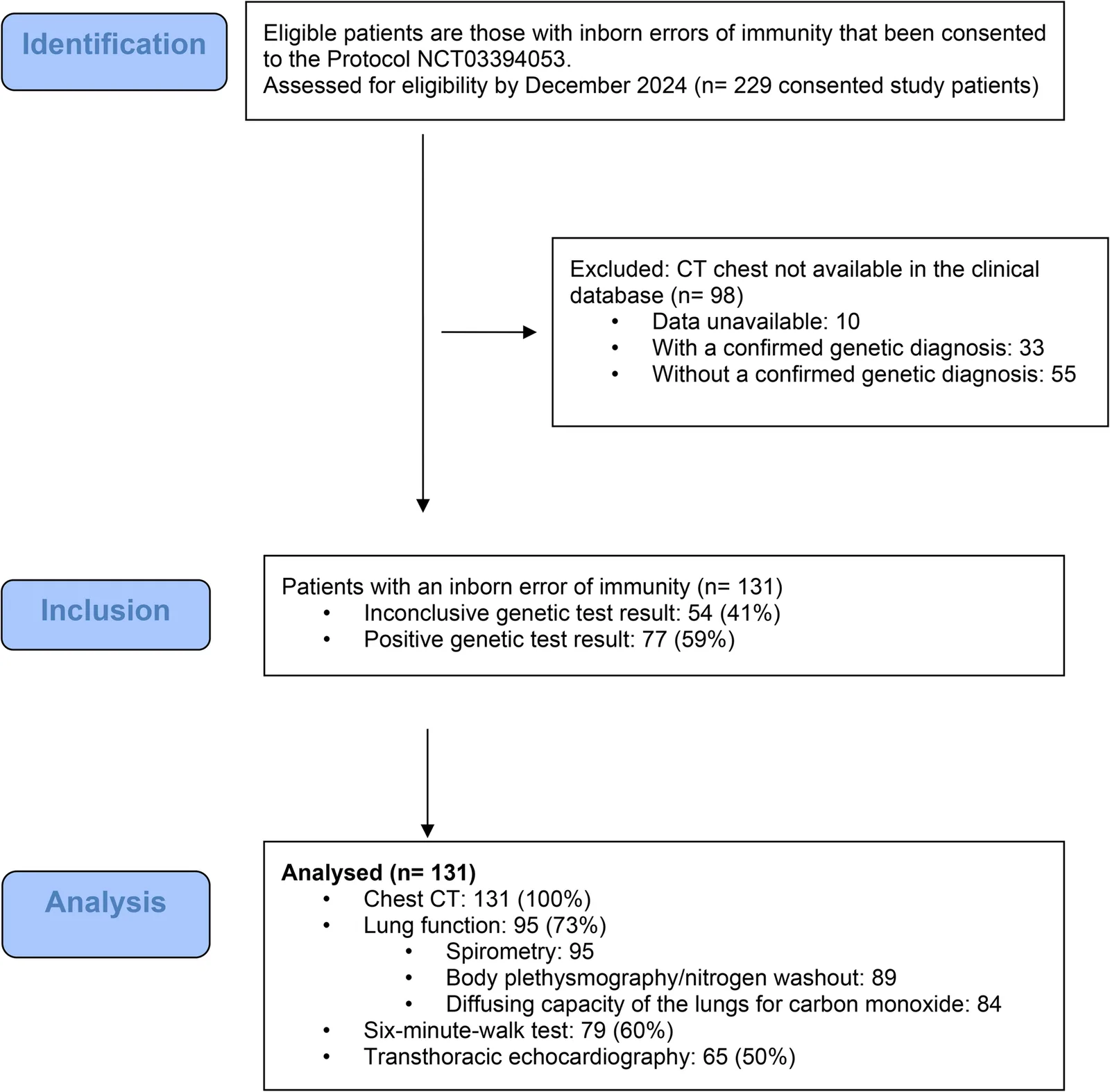
STROBE flow chart showing study participant selection CT: computed tomography

As of December 2024, 229 children and adults with suspected IEIs were enrolled in the study cohort. After all exclusions (Fig. 1), 131 were included in the analysis. Among them, 60% were male, 76% were white and 30% had an immunodeficiency affecting cellular and humoral immunity. 33% (43/131) of the patients were unclassified. Thirty-one distinct IEIs were identified based on a genetic diagnosis. The most common genetic variant identified in this cohort was in *RAG1* 14% (18/131). We report the variants most likely to underlie the patient’s disease, including heterozygosity for genes such as *CFTR* that may increase susceptibility to respiratory disease. Patients with *TNFSF13B* variants lacked alternative pathogenic variants to explain their phenotype. The median age of the patients at first chest CT was 20.3 (IQR: 9.9–33) years. Selected characteristics of the study population are shown in Table 1.

**Table 1:** Selected patient characteristics, overall and by bronchiectasis status

	All patients (*N* = 131)	Bronchiectasis (*n* = 35)	No Bronchiectasis (*n* = 96)	*P*-value
Age at first chest CT (years), median (IQR)	20.3 (9.9–33.0)	25.5 (13.3–38.0)	17.6 (9.0–30.4)	0.026*
Children i.e. 0–17 years, n (%)	54 (41.2)	11 (31.4)	43 (44.8)	0.240
Male, n (%)	79 (60.3)	18 (51.4)	61 (63.5)	0.293
Continent, n (%)				0.407
Africa (Algeria, Egypt)	12	4 (11.4)	8 (8.3)	
Asia (Kuwait, Lebanon, Saudi Arabia)	4	1 (2.9)	3 (3.1)	
Europe (Croatia, France, Greece, Italy, Lithuania)	15	7 (20.0)	8 (8.3)	
North America (Canada, USA, Cost Rica)	89	20 (57.1)	69 (71.9)	
Oceania (New Zealand)	2	0 (0.0)	2 (2.1)	
South America (Colombia, Peru, Venezuela)	9	3 (8.6)	6 (6.2)	
Race, n (%)				0.480
American Indian/Alaska Native	1 (0.8)	0 (0.0)	1 (1.0)	
Asian	9 (6.9)	3 (8.6)	6 (6.2)	
Black	2 (1.5)	1 (2.9)	1 (1.0)	
Multiracial	7 (5.3)	3 (8.6)	4 (4.2)	
Native Hawaiian/Pacific Islander	3 (2.3)	0 (0.0)	3 (3.1)	
Another race	2 (1.5)	1 (2.9)	1 (1.0)	
Unknown	8 (6.1)	4 (11.4)	4 (4.2)	
White	99 (75.6)	23 (65.7)	76 (79.2)	
IUIS classification of IEIs, n (%)				0.083
I. Immunodeficiencies affecting cellular and humoral immunity	39(29.8)	14 (40.0)	25 (26.0)	
II. Combined immunodeficiencies with associated or syndromic features	15 (11.5)	1 (2.9)	14 (14.6)	
III. Predominantly antibody deficiencies	20 (15.3)	9 (25.7)	11 (11.5)	
IV. Diseases of immune dysregulation	7 (5.3)	2 (8.6)	4 (4.2)	
V. Congenital defects of phagocyte number or function	0 (0)	0 (0)	0 (0)	
VI. Defects of intrinsic and innate immunity	4 (3.1)	1 (2.9)	3 (3.1)	
VII. Autoinflammatory disorders	0 (0)	0 (0)	0 (0)	
VIII. Complement deficiencies	1 (0.8)	1 (1.0)	0 (0)	
IX. Bone marrow failure	0 (0)	0 (0)	0 (0)	
X. Phenocopies of inborn errors of immunity	0 (0)	0 (0)	0 (0)	
Unclassified	43 (32.8)	7 (20.0)	36 (37.5)	
Other genetic disease	2 (1.5)	0 (0)	2 (2.1)	
Specific pathogenic variant or chromosomal abnormality, n (%)				0.130
22q11.21	1 (0.8)	0 (0)	1 (1.0)	
*BTK*	1 (0.8)	0 (0)	1 (1.0)	
*CD3G*	1 (0.8)	0 (0)	1 (1.0)	
*CD40LG*	7 (5.3)	0 (0)	7 (7.3)	
CFH	1 (0.8)	0 (0)	1 (1.0)	
*FOXN1*	3 (2.3)	0 (0)	3 (3.1)	
*GTF3A*	1 (0.8)	1 (2.9)	0 (0)	
*IFNGR1*	1 (0.8)	0 (0)	1 (1.0)	
*IKBKG*	1 (0.8)	0 (0)	1 (1.0)	
*IKZF3*	1 (0.8)	1 (2.9)	0 (0)	
*IL2RG*	4 (3.1)	2 (5.7)	2 (2.1)	
*IL7*	1 (0.8)	0 (0)	1 (1.0)	
*NFKB1*	1 (0.8)	0 (0)	1 (1.0)	
*NFKB2*	1 (0.8)	0 (0)	1 (1.0)	
*PDHA1*	1 (0.8)	0 (0)	1 (1.0)	
*PIK3CD*	1 (0.8)	1 (2.9)	0 (0)	
*PIK3R1*	2 (1.5)	1 (2.9)	1 (1.0)	
*PNP*	1 (0.8)	0 (0)	1 (1.0)	
*PTPN11*	1 (0.8)	0 (0)	1 (1.0)	
*RAG1*	17 (13.0)	9 (25.7)	8 (8.3)	
*RAG1* + CF carrier	1 (0.8)	1 (2.9)	0 (0)	
*RAG2*	1 (0.8)	1 (2.9)	0 (0)	
*RASGRP1*	1 (0.8)	0 (0)	1 (1.0)	
*SASH3*	5 (3.8)	0 (0)	5 (5.2)	
*STAT1GOF*	6 (4.6)	3 (8.6)	3 (3.1)	
*STAT3GOF*	1 (0.8)	0 (0)	1 (1.0)	
*TNFSRF13B*	3 (2.3)	0 (0)	3 (3.1)	
*TPP2*	1 (0.8)	0 (0)	1 (1.0)	
*TTC7A*	1 (0.8)	0 (0)	1 (1.0)	
*WAS*	7 (5.3)	0 (0)	7 (7.3)	
*XIAP*	1 (0.8)	0 (0)	1 (1.0)	
*ZAP70*	1 (0.8)	1 (2.9)	0 (0)	
Uncertain	54 (41.2)	14 (40.0)	40 (41.7)	

*P*-values by *Wilcoxon-Mann-Whitney test for continuous variables that are not normally distributed, and chi-squared for binary or categorical variables

*IEIs*: inborn errors of immunity; *IUIS*: International Union of Immunological Societies expert committee’s updated 2024 classification of human inborn errors of immunity; Uncertain genetic diagnoses were documented in patients who underwent genetic testing by whole genome/exome sequencing, but the results could not explain the clinical phenotype

### Bronchiectasis Prevalence

**Fig. 2:**
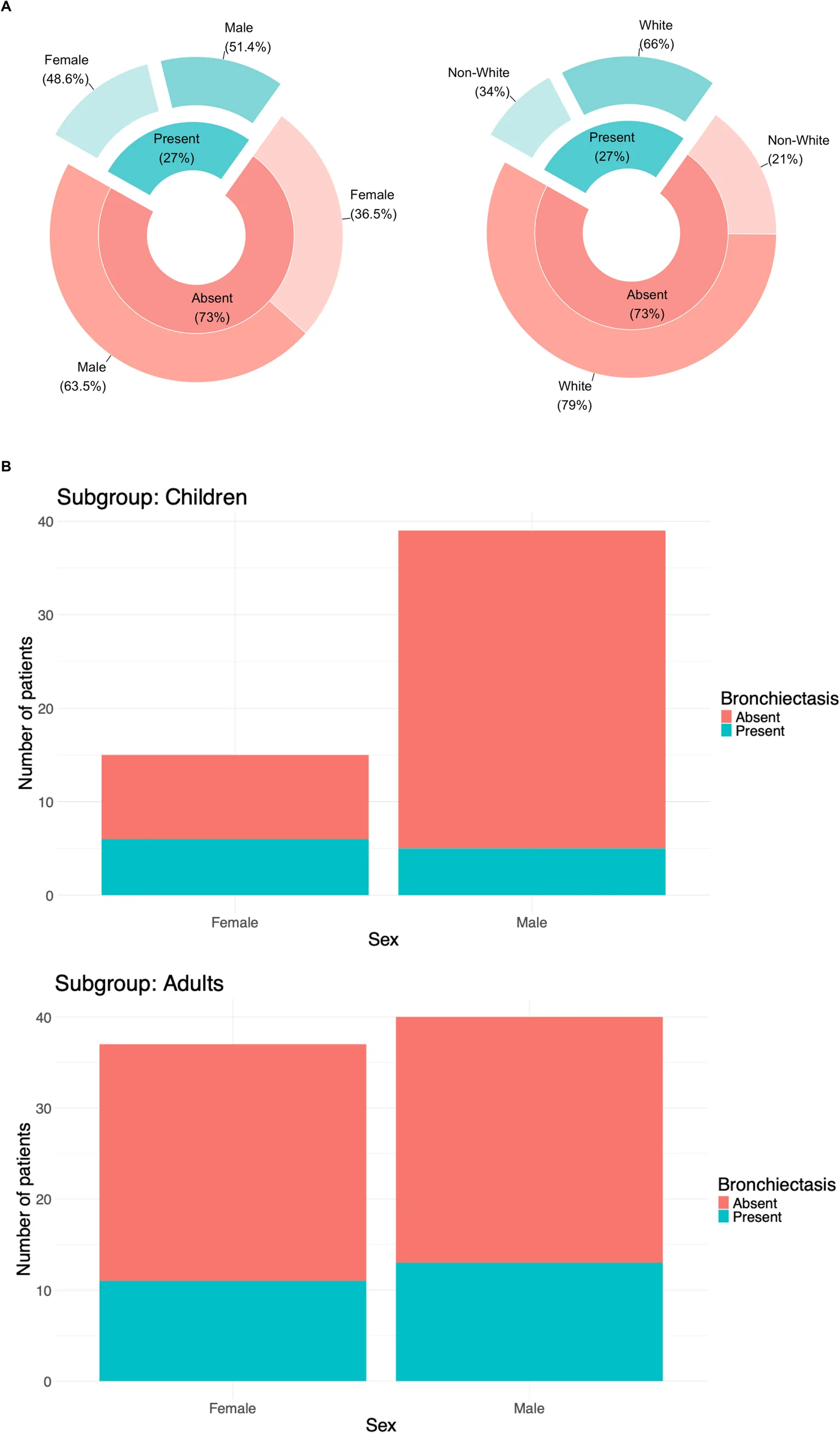

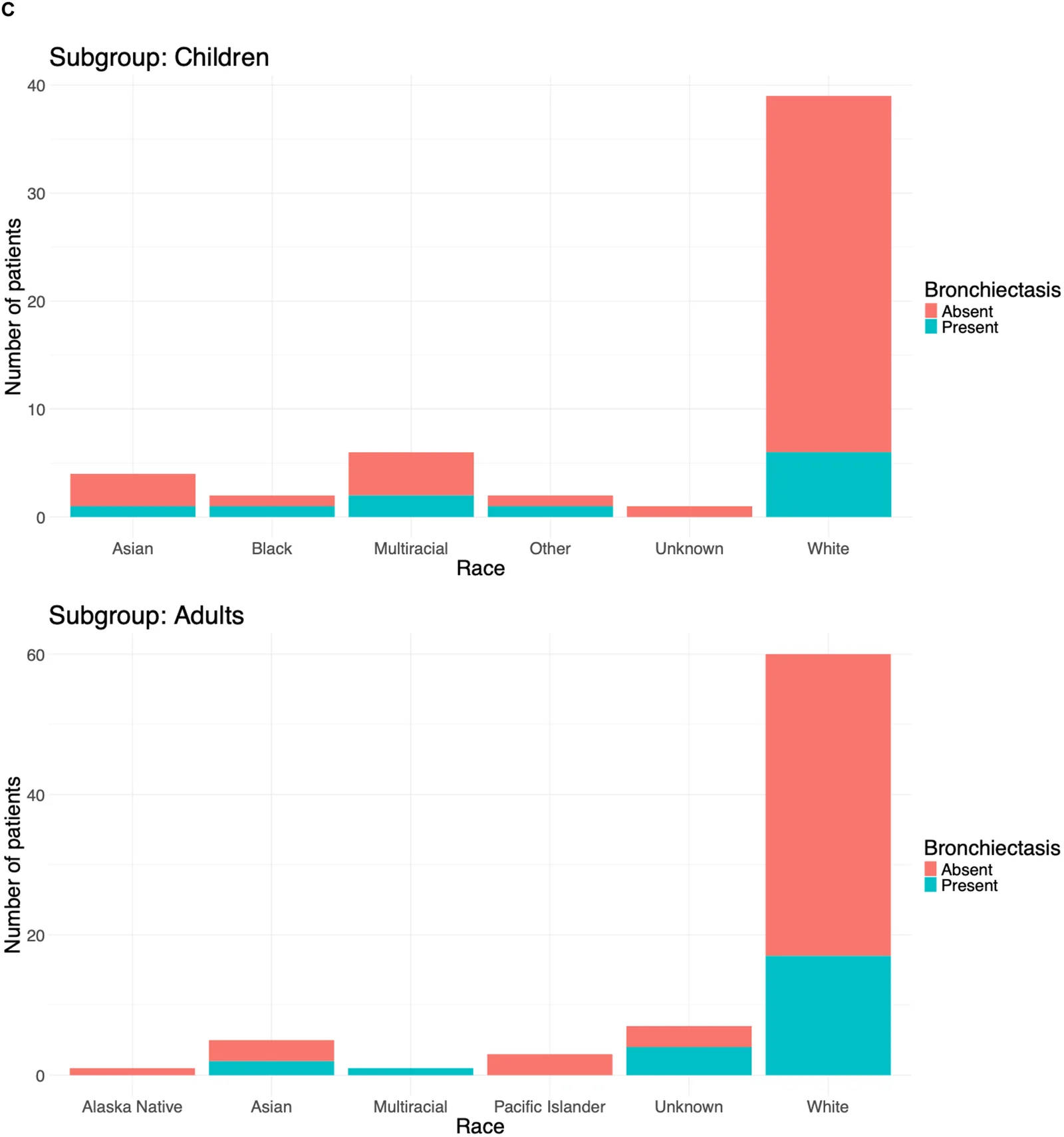

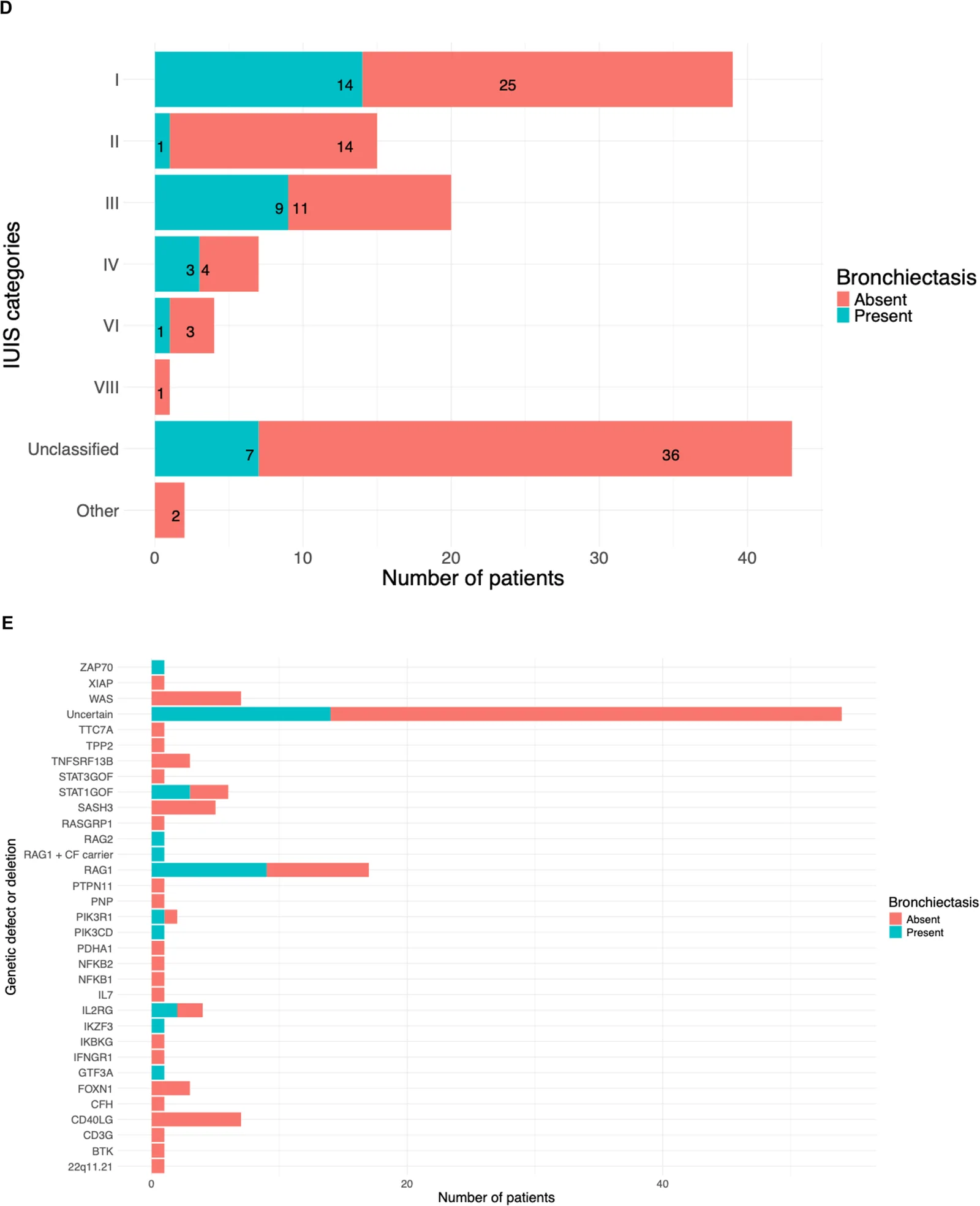
Bronchiectasis in relation to sex, race, IUIS classification and genetic mutations. **A** Nested pie charts describing the prevalence of bronchiectasis by sex and race. **B** Bar graph illustrating the bronchiectasis status of patients by sex in children and adults. **C** Bar graph illustrating the bronchiectasis status of patients by specific race in children and adults. **D** Bar graph visualizing the bronchiectasis status of patients by the IUIS classification. (I) Immunodeficiencies affecting cellular and humoral immunity, (II) Combined immunodeficiencies with associated or syndromic features, (III) Predominantly antibody deficiencies, (IV) Diseases of immune dysregulation, VI. Defects of intrinsic and innate immunity, VII. Autoinflammatory disorders, IX. Bone marrow failure, X. Phenocopies of inborn errors of immunity associated with autoantibodies or somatic variants, Unclassified, Other non-IEI genetic disorders. **E** Bar graph depicting the proportion of patients with specific chromosomal abnormalities or pathogenic variants among those with and without bronchiectasis

The prevalence of bronchiectasis in this cohort was 27% (95%CI, 19.9–34.9). Eighteen out of 35 patients with bronchiectasis (51%) were male, 11 (31%) were children, 20 (57%) were North American, and 23 (66%) were white. Fourteen (40%) were classified having an immunodeficiency affecting cellular and humoral immunity, 29% had biallelic *RAG1* pathogenic variants and 20% were unclassified. The median age at first chest CT for patients with bronchiectasis was 25.5 (IQR: 13.3–38.0) years, significantly older than patients without bronchiectasis, 17.6 (IQR: 9.0–30.4) years, *P* = 0.026 (Table 1; Fig. 2).

### Bronchiectasis and other Chest CT Findings

**Fig. 3:**
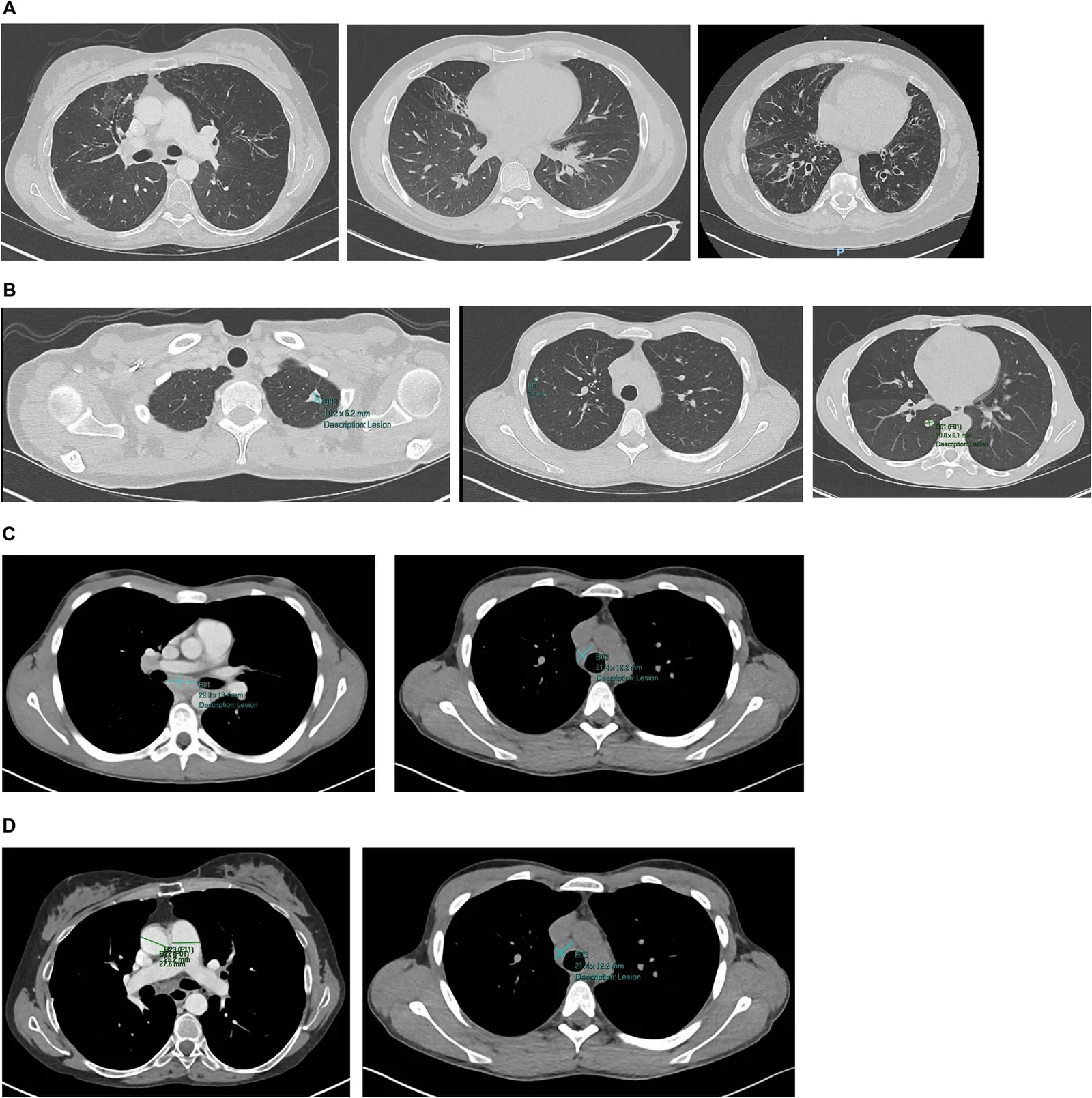
Selected chest computed tomography to illustrate pertinent findings. **A** Lung window axial projections: (i) Upper lobes: Bilateral bronchiectasis predominantly of the varicose type. Patchy ground glass and tree in bud opacities bilaterally. Findings in a 37-year-old female patient with severe combined immunodeficiency (SCID), *RAG1*.; (ii) Right middle lobe and lingula: Predominantly focal right middle lobe bronchiectasis. Mosaic attenuation indicating air trapping in the right middle lobe and lingula noted on expiration. Some interlobular septal thickening and tree in bud opacities bilaterally. Findings in a 31-year-old male patient with a predominantly antibody deficiency, *PIK3CD*. (iii) Lower lobes: Diffuse bronchiectasis. Note the broncho-arterial ratios are significantly > 1. Some areas with varicose bronchiectasis. Bilateral interlobular septal thickening, tree in bud and ground glass opacities. Findings in a 41-year-old male patient with SCID, *IL2RG*. **B** Lung window axial projections: (i) Apices: Peribronchovascular nodule in the left apex measuring 1 × 0.6 cm and contains a bronchiectactic airway within it. Findings in a 38-year-old female patient *NEMO* carrier classified as an uncertain genetic mutation; (ii) Upper lobes: Small lung nodule measuring 0.4 cm in the right upper lobe. Findings in a 20-year-old male patient with SCID, due to *RAG1biallelic variants*.; (iii) Lower lobes: Lung nodule measuring 1 × 0.6 cm in the right lower lobe. Findings in a 25-year-old male patient with an innate immune defect resulting in susceptibility to herpes simplex encephalitis and T cell development impairment, *GTF3A*. **C** Lung window axial projections: (i) With intravenous contrast: Multiple prominent mediastinal lymph nodes. Subcarinal lymphadenopathy highlighted; (ii) Without intravenous contrast: Multiple prominent mediastinal lymph nodes. Right paratracheal lymphadenopathy highlighted. Findings in a 20-year-old patient with SCID, *RAG1*; **D** Lung window axial projections: With intravenous contrast: Diameter of the aorta and pulmonary trunk appear equal; ii) Without intravenous contrast: Six months later, the diameter of the pulmonary trunk appears much larger than the aorta, suggestive of pulmonary hypertension, which was confirmed on echocardiography. Findings in a 39-year-old patient with SCID, *RAG1*

The median number of chest CT scans in patients with bronchiectasis was 3.0 (1–6.5), significantly greater than in patients without bronchiectasis 1.0 (1-2), *P* = 0.003 (Table 2). The distribution of bronchiectasis varied from focal, multi-lobar and diffuse lung involvement. Similarly, different patterns of bronchiectasis were identified including varicose, cylindric and cystic types as illustrated in selected cases (Fig. 3).

**Table 2:** Chest computed tomography characteristics, overall and by bronchiectasis status

	All patients (*N* = 131)	Bronchiectasis (*n* = 35)	No Bronchiectasis (*n* = 96)	*P*-value
Number of CT chests, median (IQR)	1.0 (1.0–3.0)	3.0 (1.0-6.5)	1.0 (1.0–2.0)	0.003*
CT chest finding, n (%)				
*Airway*				
Tree in bud	17 (13.0)	13 (37.1)	4 (4.2)	< 0.001
Mucous plugging	11 (8.4)	8 (22.9)	3 (3.1)	0.001
Mosaic attenuation	9 (6.9)	4 (11.4)	5 (5.2)	0.392
Bronchiolitis obliterans	1 (0.8)	0 (0)	1 (1.0)	1.000
*Parenchyma*				
Nodules	72 (55.0)	29 (82.9)	43 (44.8)	0.001
Ground glass opacification	52 (39.7)	21 (60.0)	31 (32.3)	0.008
Atelectasis	38 (29.0)	14 (40.0)	24 (25.0)	0.145
Airspace opacification	29 (22.1)	12 (34.3)	17 (17.7)	0.074
Fibrosis	27 (20.6)	13 (37.1)	14 (14.6)	0.010
Calcification	18 (13.7)	9 (25.7)	9 (9.4)	0.034
Reticular pattern	10 (7.6)	4 (11.4)	6 (6.2)	0.538
Interlobular septal thickening	5 (3.8)	3 (8.6)	2 (2.1)	0.230
Cavity	5 (3.8)	3 (8.6)	2 (2.1)	0.230
Mass	4 (3.1)	0 (0)	4 (4.2)	0.514
Cyst	2 (1.5)	0 (0.0)	2 (2.1)	0.956
Emphysema	2 (1.5)	1 (2.9)	1 (1.0)	1.000
*Pleura*				
Pleural effusion	12 (9.2)	6 (17.1)	6 (6.2)	0.116
*Mediastinum*				
Lymphadenopathy	52 (40.0)	18 (52.9)	34 (35.4)	0.112
*Vasculature*				
Pulmonary hypertension	6 (4.6)	2 (6.1)	4 (4.5)	1.000
*Radiological features suggestive of GLILD*				
GLILD-associated triad of pulmonary nodules, ground glass opacities and mediastinal/hilar lymphadenopathy	21 (16.0)	10 (28.6)	11 (11.5)	0.036

*CT*: computed tomography; *GLILD*: granulomatous-lymphocytic interstitial lung disease

*P*-values by *Wilcoxon-Mann-Whitney test for continuous variables that are not normally distributed, and chi-squared for binary or categorical variables

**Fig. 4:**
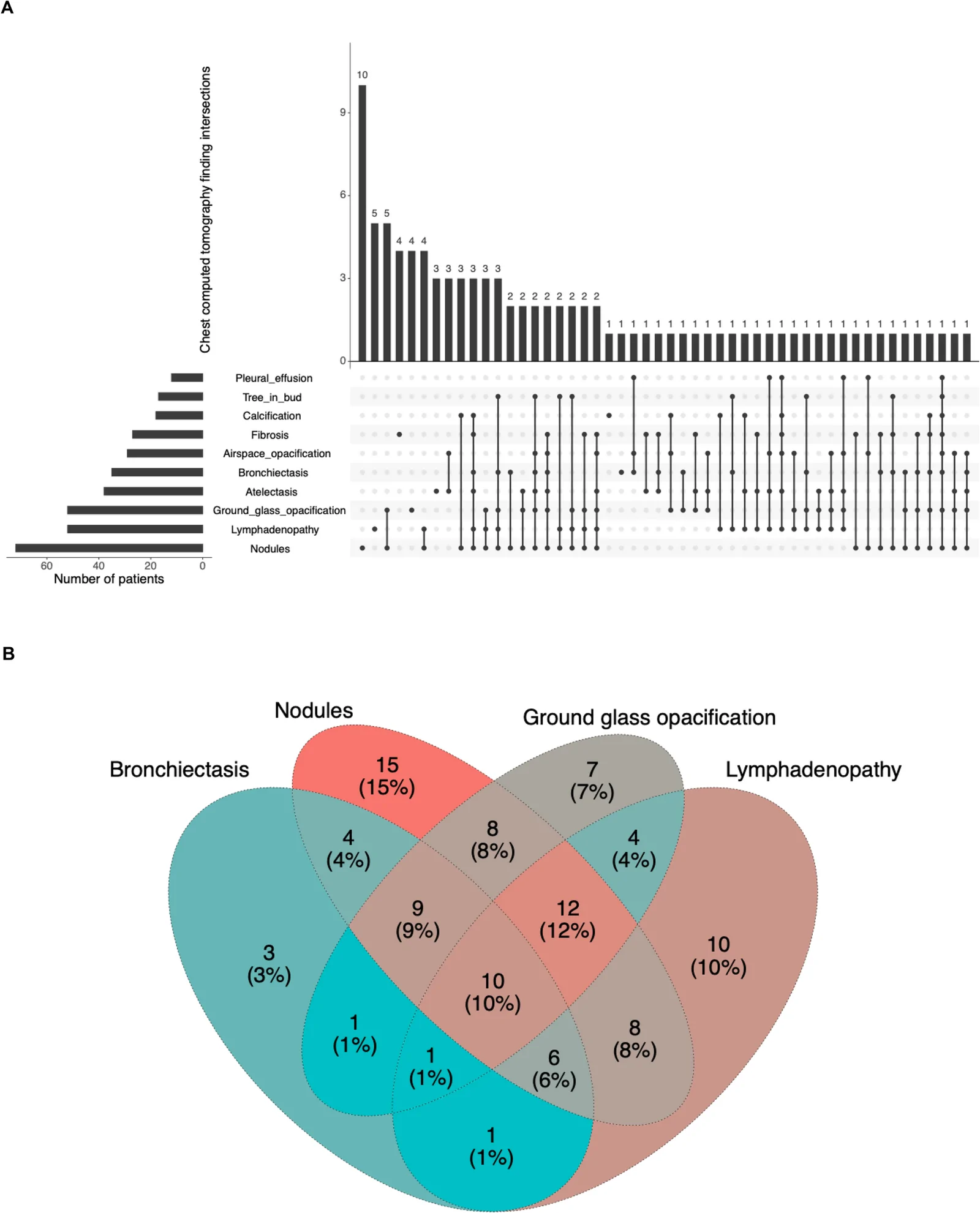
The frequency, intersection and chest computed tomography findings of patients with inborn errors of immunity. **A** An Upset plot visualizing the intersection between the ten most common chest computed tomography findings among 131 participants. **B** A Venn diagram showing bronchiectasis in relation to the three most common chest computed tomography findings: nodules, lymphadenopathy and ground glass opacification

The airways, parenchyma, pleura, mediastinum and vasculature were involved in patients with IEIs, with significant overlap of findings (Table 2; Figs. 3 and 4). Nodules (55%), ground glass opacification (40%) and lymphadenopathy (40%) were the three most common chest CT findings (Fig. 4). A clinical-radiological pattern consistent with GLILD was identified in 21 of 131 patients (16%), including 10 of 35 patients with bronchiectasis (29%). Tree in bud, mucous plugging, nodules, ground glass opacification, fibrosis, calcification and GLILD were identified on chest CT more frequently among patients with bronchiectasis compared to their IEI counterparts without bronchiectasis, *P* < 0.001, *P* = 0.01, *P* = 0.001, *P* = 0.008, *P* = 0.010, *P* = 0.034, *P* = 0.036 respectively (Table 2).

### Predictors of Bronchiectasis

Overall, age at first chest CT (aOR 1.04, 1.01–1.08, *P* = 0.017); immunodeficiencies affecting cellular and humoral immunity (aOR 4.30, 1.42–14.54, *P* = 0.013), predominantly antibody deficiencies (aOR 5.83, 1.66–22.26, *P* = 0.007) and diseases of immune dysregulation (aOR 7.56, 1.07–52.72, *P* = 0.037) were significant positive predictors of bronchiectasis independent of sex and race. Subgroup analyses revealed that female sex (aOR 5.77, 1.09–35.25, *P* = 0.043) was a significant positive predictor of bronchiectasis among children independent of race and IUIS class, whereas predominantly antibody deficiencies (aOR 6.92, 1.59–34.29, *P* = 0.012) was a significant positive predictor of bronchiectasis among adults independent of sex and race. A trend towards immunodeficiencies affecting cellular and humoral immunity (aOR 3.77, 0.99–15.73, *P* = 0.056) positively predicting bronchiectasis was observed among adults (Tables 3, 4 and 5).

**Table 3:** Predictors of bronchiectasis among children and adults with inborn errors of immunity

	*Adjusted Odds Ratio (95% CI)	*P*-value
Age at first chest CT scan	1.04 (1.01, 1.08)	0.017†
Male (Reference: Female)	0.82 (0.32, 2.08)	0.667
White (Reference: Non-White)	0.48 (0.18, 1.27)	0.136
IUIS classification (Reference: Unclassified)		
I. Immunodeficiencies affecting cellular and humoral immunity	4.30 (1.42, 14.54)	0.013†
II. CID with associated or syndromic features (predominantly WAS)	4.06 (0.02, 2.90)	0.440
III. Predominantly antibody deficiency	5.83 (1.66, 22.26)	0.007‡
IV. Diseases of immune dysregulation	7.56 (1.07, 52.72)	0.037†
VI. Defects of intrinsic and innate immunity	2.20 (0.09, 24.95)	0.549
VIII. Complement deficiencies	9.06e-08 (NA, Inf)	0.995
Other non-IEI genetic disorder	8.79e-07 (NA, 7.63e + 205)	0.993

*Adjusted for age, sex, race and IUIS class

§Trend towards positively predicting bronchiectasis: *P*-value <0.1 and >0.05

†Statistically significant positive predictor of bronchiectasis: *P*-value <0.05 and >0.001

‡Statistically significant positive predictor of bronchiectasis: *P*-value <0.001

*CID*: Combined immunodeficiency; *IEI*: inborn errors of immunity; *Inf*: infinity; *NA*: not available; *IUIS*: International Union of Immunological Societies; *WAS*: Wiskott Aldrich Syndrome

**Table 4:** Predictors of bronchiectasis among children with inborn errors of immunity

	*Adjusted Odds Ratio (95% CI)	*P*-value
Female (Reference: Male)	5.77 (1.09, 35.25)	0.043†
White (Reference: Non-White)	0.27 (0.05, 1.28)	0.103
IUIS classification (Reference: Unclassified)		
I. Immunodeficiencies affecting cellular and humoral immunity	1.95 (0.28, 17.90)	0.513
II. CID with associated or syndromic features (predominantly WAS)	4.37e-08 (NA, 2.40e + 66)	0.994
III. Predominantly antibody deficiency	2.31 (0.18, 29.24)	0.550
IV. Diseases of immune dysregulation	2.00 (0.16, 26.62)	0.582
VI. Defects of intrinsic and innate immunity	5.65e-08 (NA, 1.58e + 233)	0.997
VIII. Complement deficiencies	2.10e-08 (NA, Inf)	0.998
Other non-IEI genetic disorder	4.18e-08 (NA, 9.39e + 223)	0.997

*Adjusted for age, sex, race and IUIS class

§Trend towards positively predicting bronchiectasis: *P*-value < 0.1 and > 0.05

†Statistically significant positive predictor of bronchiectasis: *P*-value < 0.05 and > 0.001

‡Statistically significant positive predictor of bronchiectasis: *P*-value < 0.001

*CID*: Combined immunodeficiency; *IEI*: inborn errors of immunity; *Inf*: infinity; *NA*: not available; *IUIS*: International Union of Immunological Societies; *WAS*: Wiskott Aldrich Syndrome

**Table 5:** Predictors of bronchiectasis among adults with inborn errors of immunity

	*Adjusted Odds Ratio (95% CI)	*P*-value
Male (Reference: Female)	0.82 (0.32, 2.08)	0.581
White (Reference: Non-White)	0.48 (0.18, 1.27)	0.577
IUIS classification (Reference: Unclassified)		
I. Immunodeficiencies affecting cellular and humoral immunity	3.77 (0.99, 15.73)	0.056§
II. CID with associated or syndromic features (predominantly WAS)	0.65 (0.03, 5.38)	0.721
III. Predominantly antibody deficiency	6.92 (1.59, 34.29)	0.012†
VI. Defects of intrinsic and innate immunity	4.09 (0.14, 118.79)	0.352

*Adjusted for age, sex, race and IUIS class

§Trend towards positively predicting bronchiectasis: *P*-value <0.1 and >0.05

†Statistically significant positive predictor of bronchiectasis: *P*-value <0.05 and >0.001

‡Statistically significant positive predictor of bronchiectasis: *P*-value <0.001

*CID*: Combined immunodeficiency; *IEI*: inborn errors of immunity; *Inf*: infinity; *NA*: not available; *IUIS*: International Union of Immunological Societies; *WAS*: Wiskott Aldrich Syndrome

### Cardiopulmonary Complications

**Fig. 5:**
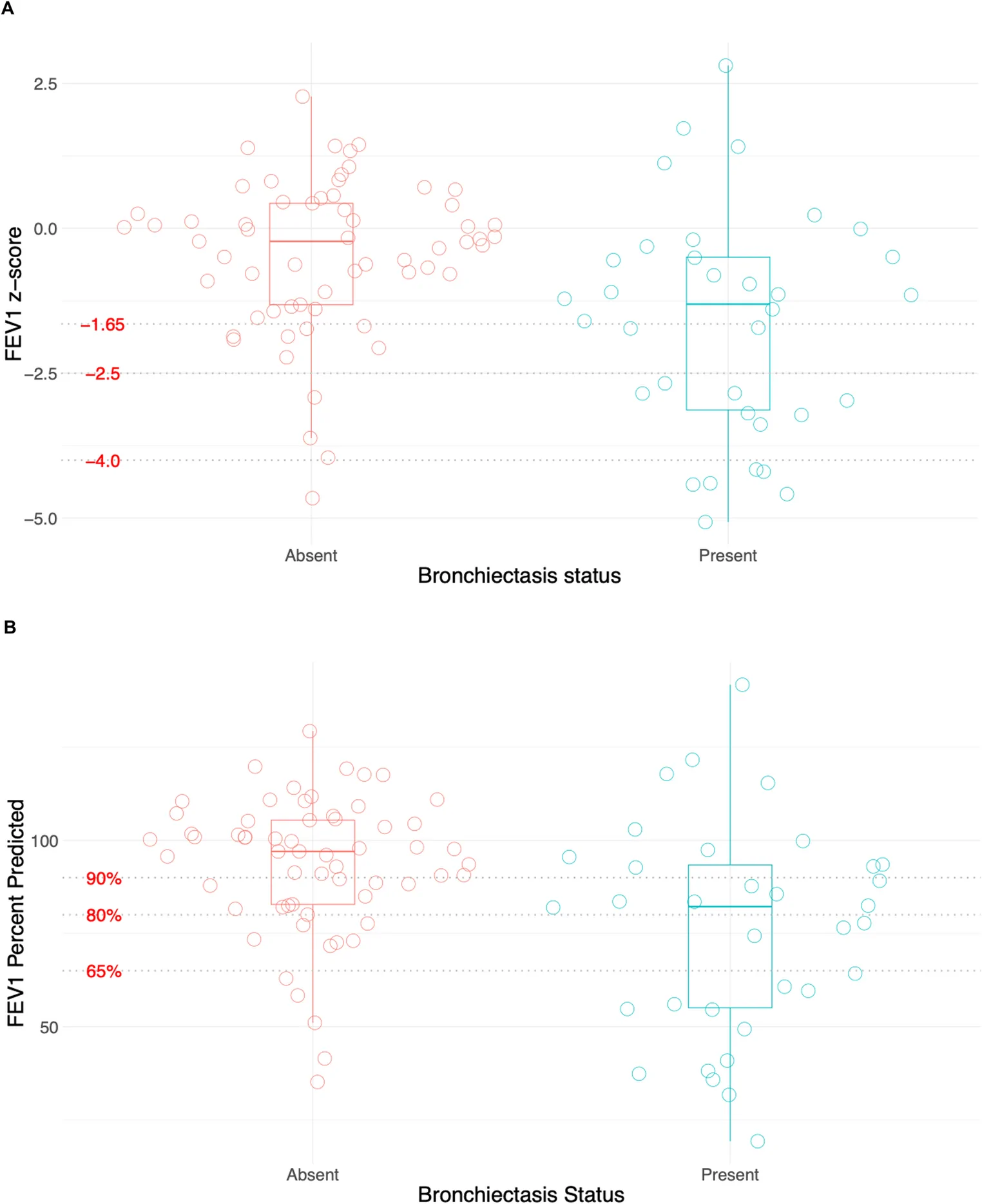

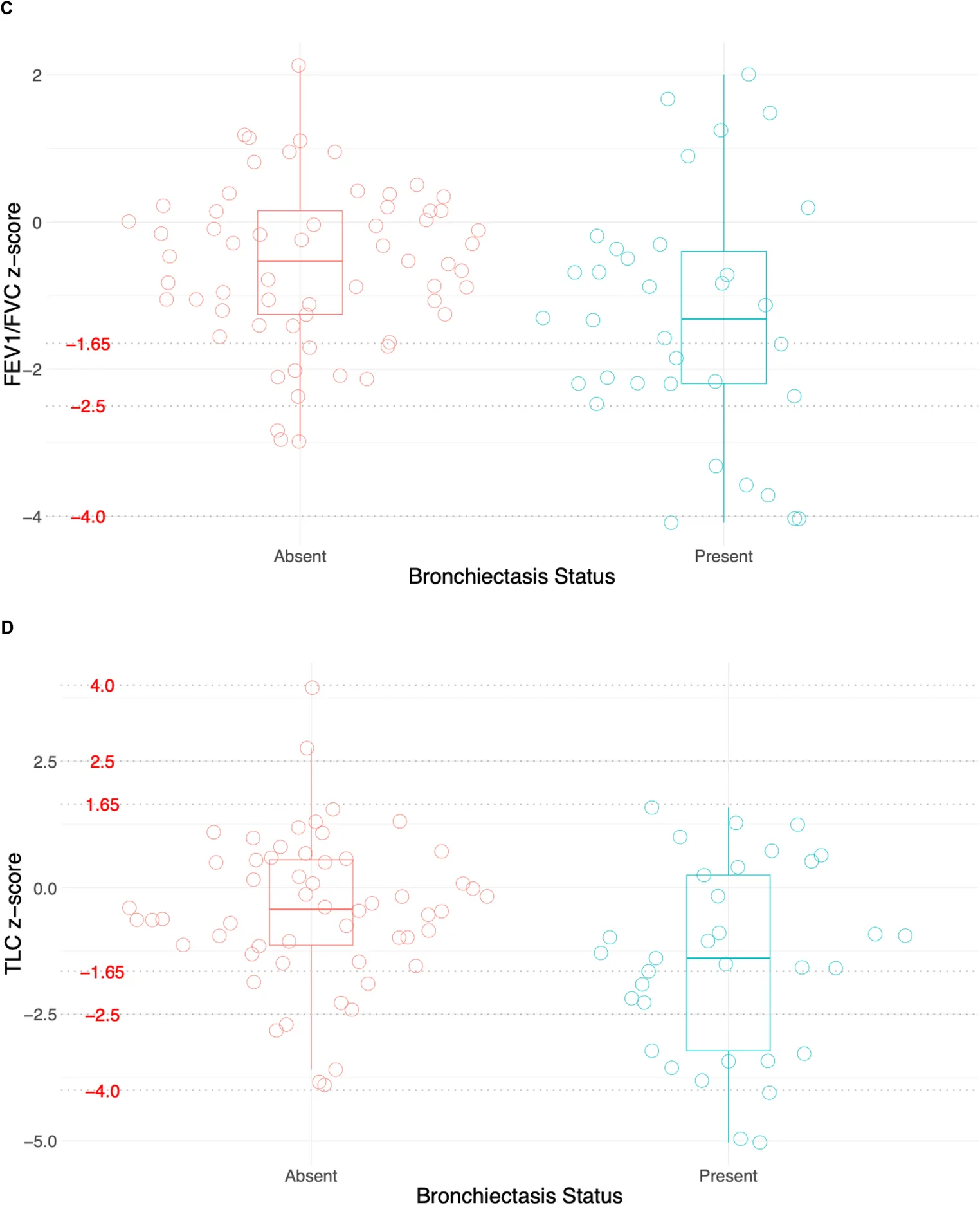

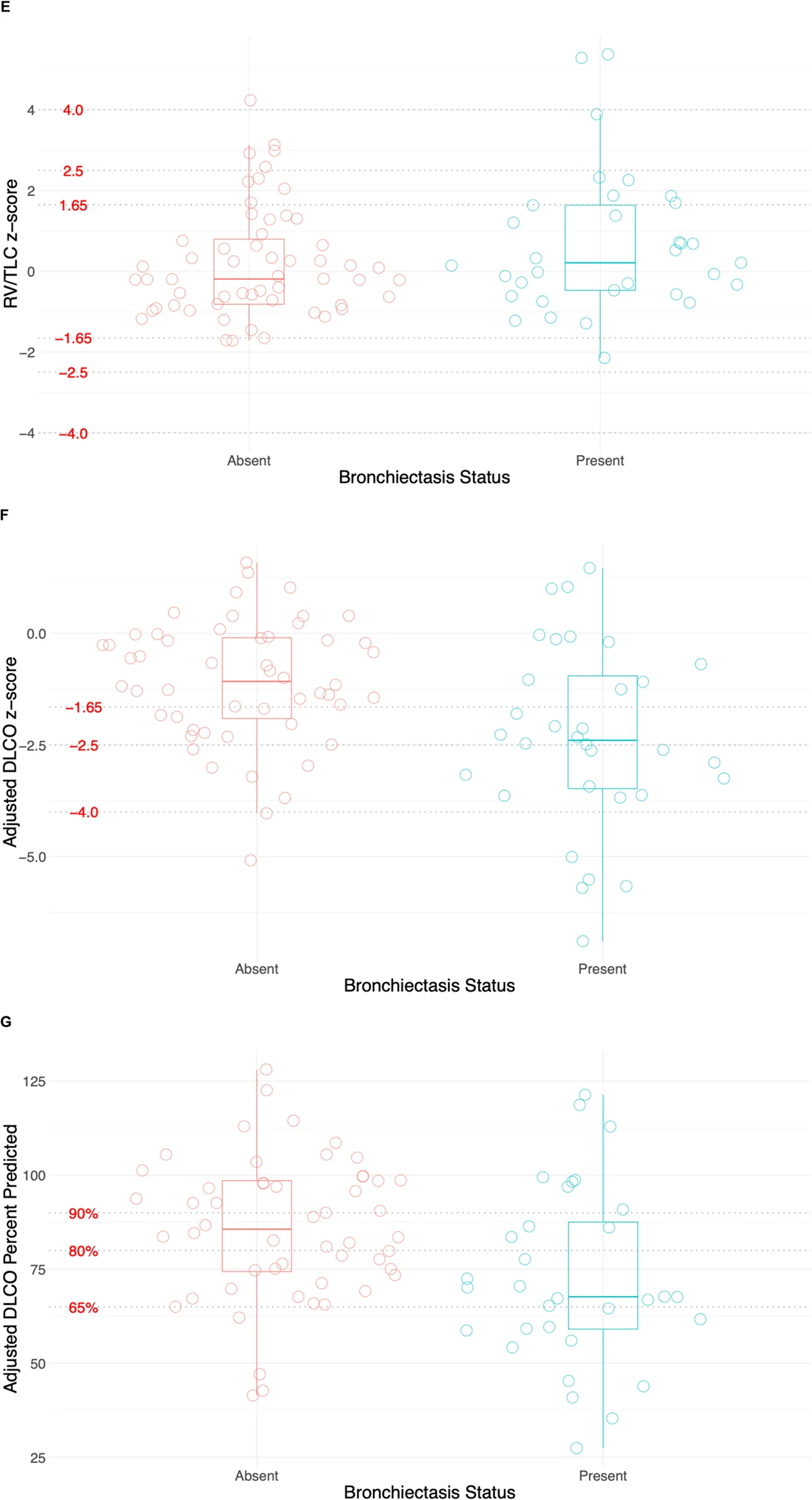
Box and Sina plots depicting several pulmonary function test parameters by bronchiectasis status included in the pulmonary-specific Hematopoietic Cell Transplantation Comorbidity Index and the 2021 American Thoracic Society/European Respiratory Society Technical Standard: **A** FEV_1_ z-scores; **B** FEV_1_ percent predicted values; **C** FEV_1_/FVC z-scores for the assessment of airflow obstruction; **D** TLC z-score for the assessment of restriction; **E** RV/TLC z-score for the assessment of hyperinflation; **F** adjusted DLCO z-score for the assessment of gas transfer impairment; and (**G**) adjusted DLCO percent predicted

**Fig. 6:**
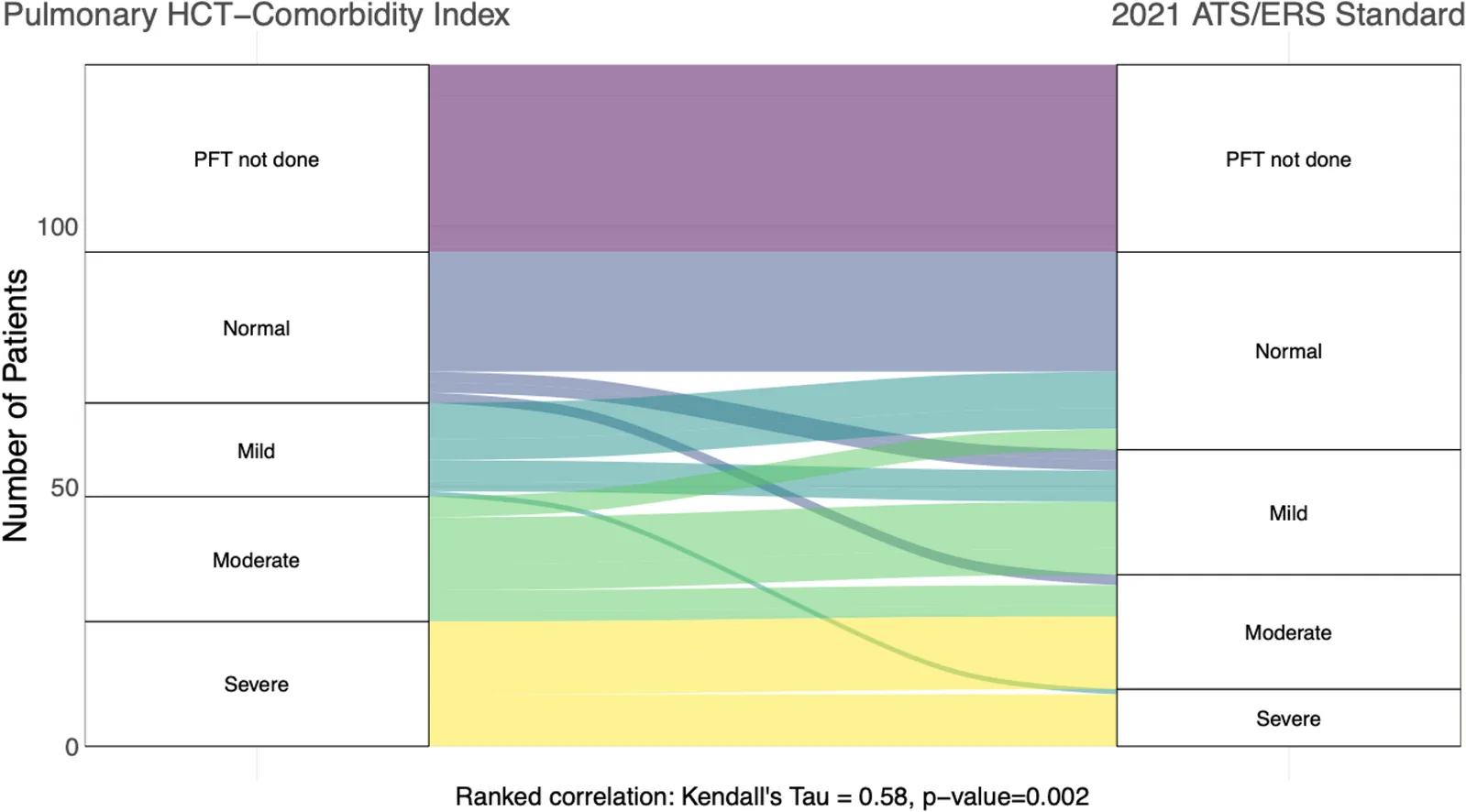
Alluvial plot illustrating the classification of pulmonary physiological impairment among inborn error of immunity patients based on parameters used in the pulmonary-specific Hematopoietic Cell Transplantation Comorbidity Index and the 2021 American Thoracic Society/European Respiratory Society Technical Standard. The Kendall rank correlation coefficient and p-value assessing the degree of association between these two ranked classification systems shows a significant moderate positive correlation

Cardiopulmonary complications, specifically abnormalities in spirometry, lung volume, DLCO, six-minute walk testing and pulmonary pressures were significantly greater among patients with bronchiectasis compared to those without bronchiectasis, *P* < 0.001 (Table 6; Fig. 5). A moderate and significant positive correlation was observed between pulmonary function severity classifications derived from pulmonary-specific Hematopoietic Cell Transplant comorbidity index (HCT-CI) [22] and the 2021 ATS/ERS physiological impairment standard [17] (Kendall’s Tau = 0.58, *P* = 0.002) (Fig. 6). There were 5/35 (15%) deaths in patients with bronchiectasis and 3/96 (0.03%) deaths in patients without bronchiectasis, *P* = 0.046 (Table 6).

**Table 6:** Cardiopulmonary outcomes and deaths, overall and by bronchiectasis status

	All patients (*N* = 131)	Bronchiectasis (*n* = 35)	No Bronchiectasis (*n* = 96)	*P*-value
Age at last pulmonary function test, median (IQR)	23.0 (11.9–37.4)	27.6 (14.4–38.5)	20.9 (10.8–34.7)	0.073*
Number of pulmonary function tests performed, median (IQR)	1.0 (0.0–2.0)	1.0 (1.0–4.0)	1.0 (0.0–1.0)	< 0.001*
Spirometry pattern, n (%)				< 0.001
Normal	60 (45.8)	15 (42.9)	45 (46.9)	
Obstructive	16 (12.2)	6 (17.1)	10 (10.4)	
Restrictive	10 (7.6)	4 (11.4)	6 (6.2)	
Mixed	9 (6.9)	9 (25.7)	0 (0.0)	
Not done	36 (27.5)	1 (2.9)	35 (36.5)	
Lung volume pattern, n (%)				< 0.001
Normal	54 (41.2)	14 (40.0)	40 (41.7)	
Hyperinflation	12 (9.2)	5 (14.3)	7 (7.3)	
Simple restriction	18 (13.7)	11 (31.4)	7 (7.3)	
Complex restriction	2 (1.5)	1 (2.9)	1 (1.0)	
Mixed disorder	3 (2.3)	2 (5.7)	1 (1.0)	
Not done	42 (32.1)	2 (5.7)	40 (41.7)	
Diffusing capacity of the lung for carbon monoxide pattern, n (%)				< 0.001
Normal	47 (36.2)	11 (31.4)	36 (37.9)	
Abnormal	37 (28.2)	21 (60.0)	16 (16.7)	
• Pulmonary vascular abnormality [normal V_A_]	8 (6.1)	3 (8.6)	5 (5.2)	
• Localized loss of lung volume [low V_A_, high K_CO_]	0 (0)	0 (0)	0 (0)	
• Loss of alveolar capillary structure with loss of lung volume [low V_A_, low K_CO_]	29 (22.1)	18 (51.4)	11 (11.5)	
Not done	47 (36.2)	3 (8.6)	44 (46.3)	
Six-minute-walk distance, n (%)				< 0.001
< Lower limit of normal*	49 (37.4)	24 (68.6)	25 (26.0)	
Normal*	26 (19.8)	7 (20.0)	19 (19.8)	
Not done	56 (42.7)	4 (11.4)	52 (54.2)	
Pulmonary hypertension, n (%)				< 0.001
Absent	44 (35.5)	17 (51.5)	27 (29.7)	
Present	14 (11.3)	8 (24.2)	6 (6.6)	
Not done	66 (53.2)	8 (24.2)	58 (63.7)	
Deaths, n (%)	8 (6.2)	5 (14.7)	3 (3.1)	0.046

*P*-values by *Wilcoxon-Mann-Whitney test for continuous variables that are not normally distributed, and chi-squared for binary or categorical variables

GLI: Global Lung Function Initiative Global equations used; Approach follows ERS/ATS technical standards (Stanojevic et al. European Respiratory Journal. 2022) [17]; Lung volumes by body plethysmography or nitrogen washout; V_A_: alveolar volume; K_CO_: Carbon monoxide transfer coefficient; 6MWD: six minute walk distance; 6MWT: six minute walk test; SPO_2_- oxygen saturations; *Reference equations for 6MWT in healthy adults (Enright PL et al. Am J Respir Crit Care Med. 1998) [19] and healthy North African children 6–16 years old (Saad HB et al. Pediatric Pulmonology. 2009) [20]; mPAP: mean pulmonary arterial pressure; PH: pulmonary hypertension; TAPSE: tricuspid annular plane systolic excursion; TRV: tricuspid regurgitation velocity; RVOT: right ventricular outflow tract acceleration time; RVSP: right ventricular systolic pressure; ^#^estimated using the Chemla formula; *Approach follows ESC/ERS guidelines (Humbert M et al. European Respiratory Journal. 2022) [23]

### Treatment Modalities for Bronchiectasis

Eighteen out of 35 (51%) patients with bronchiectasis, received long-term azithromycin treatment, 28 (80%) got nebulized hypertonic saline with an inhaled short acting bronchodilator together with some form of airway clearance technique, 31 (89%) received intravenous (IV) or subcutaneous immunoglobulin (SCIG), and 10 (29%) had high-dose immunoglobulin therapy (IVIG ≥600 mg/kg/monthly or SCIG ≥200 mg/kg/weekly) during their management (Table 7).

**Table 7:** Treatment modalities documented for patients with bronchiectasis

	Bronchiectasis patients (*N* = 35)
Any IVIG or SCIG, *n* (%)	31 (88.6)
- High-dose IVIG or SCIG, n (%)	10 (28.6)
Airway clearance techniques and mucoactive drugs, n (%) e.g. chest percussion, postural drainage, positive expiratory pressure oscillating devices, high-frequency chest wall oscillation etc.; inhaled hypertonic saline and bronchodilator therapies	28 (80.0)
Long-term azithromycin therapy, n (%)	18 (51.4)

*IVIG*: intravenous immunoglobulin; *SCIG*: subcutaneous immunoglobulin

High-dose IVIG was ≥600 mg/kg/monthly; and high-dose SCIG ≥200 mg/kg/weekly

### Influence of a Bronchiectasis Diagnosis on Curative Treatment Decision

**Fig. 7:**
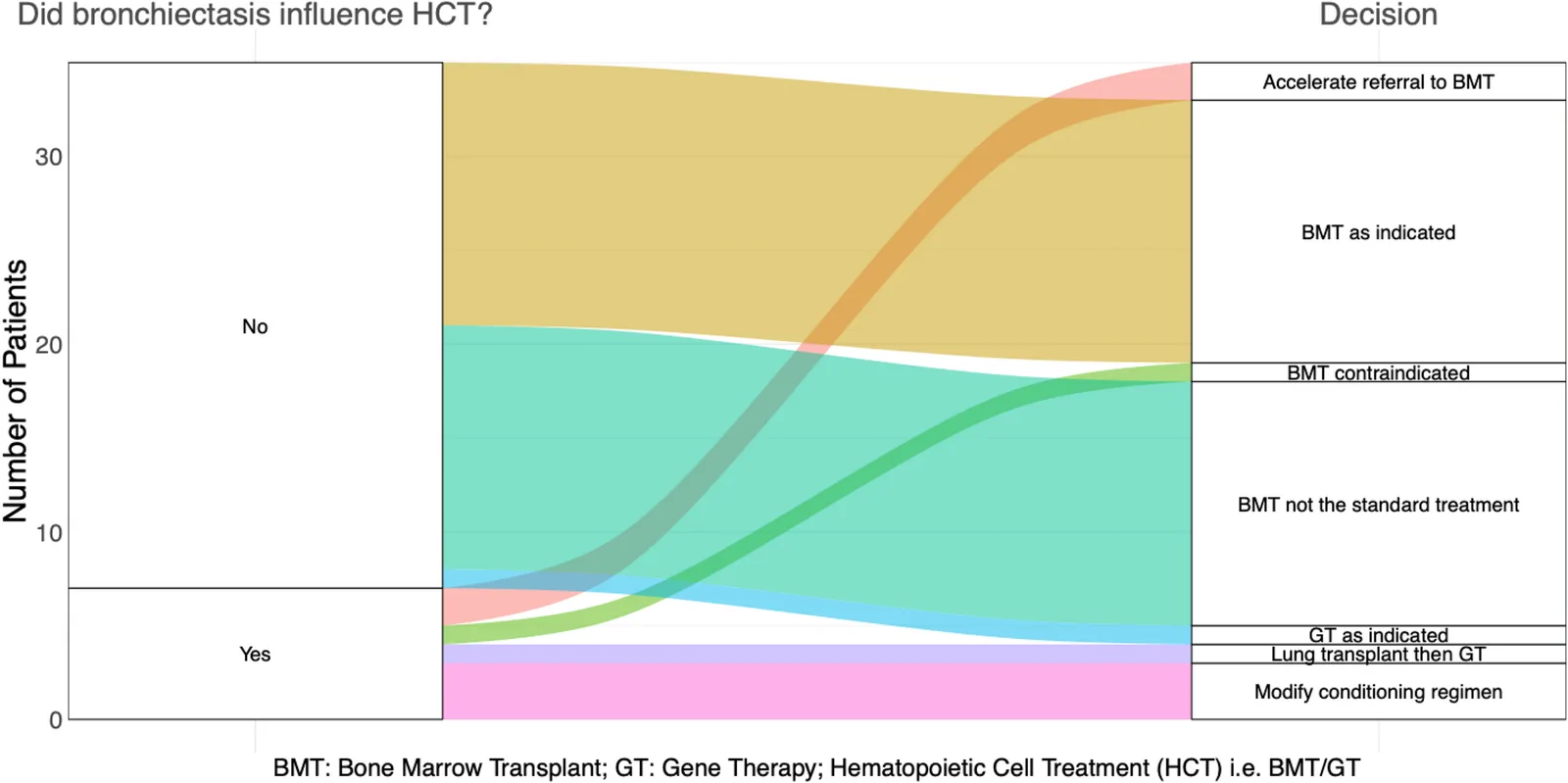
Alluvial plot depicting whether a bronchiectasis diagnosis influenced curative treatment decisions among children and adults with inborn errors of immunity

Conditions that were eligible for curative treatment such as BMT or gene therapy in this cohort comprised: deficiencies in *RAG1*, *RAG2*, *PIK3CD*, *IL2RG*, *ZAP70* and *GTF3A*; STAT1 gain of function, and severe combined immunodeficiency (SCID) phenotypes without genetic diagnosis. On the other hand, patients with common variable immunodeficiency (CVID), deficiencies in *IKZF3*, *PIK3RI*, and multiple variants of unknown significance did not qualify for BMT due to mild to moderate disease. A bronchiectasis diagnosis influenced the curative treatment in 7/35 (20%) patients (Fig. 7). BMT was contraindicated in a patient with *RAG2* deficiency due to severe bronchiectasis, based on his pulmonary-specific HCT comorbidity index. One patient with *IL2RG* deficiency received a lung transplant first and was referred for gene therapy instead of BMT. A decision to accelerate BMT was made for two patients with the following diagnoses: predominantly antibody deficiency and *RAG1* deficiency. The conditioning regimen was modified in two patients: one patient with *RAG1* deficiency by using a naked anti-c-KIT monoclonal antibody and irradiation to target CD34 + hematopoietic stem cells while avoiding busulfan toxicity on compromised lungs, and one patient with STAT1 GOF in which the busulfan dose was reduced due to her lung status. One patient with bronchiectasis and hypomorphic *IL2RG* deficiency was referred for combined lung and hematopoietic stem cell transplant (Table 8).

**Table 8:** Characteristics of inborn error of immunity patients with bronchiectasis and how the bronchiectasis diagnosis influenced curative treatment

	Genetic variants [Inheritance pattern (Phenotype)	Pulmonary specific HCT-CI	Curative treatment provided	Bronchiectasis influenced curative treatment	Curative treatment decision
1	*RAG1 * Compound Heterozygous [AR]	Severe	Yes	No	Received BMT
2	Uncertain (Unclassified)	Moderate	No	No	BMT is not the standard treatment for this IEI
3	*RAG2 *Homozygous [AR]	Severe	No	Yes	Contraindicated BMT
4	Uncertain (Predominantly antibody deficiency)	Severe	No	No	BMT is not the standard treatment for this IEI
5	Uncertain (Predominantly antibody deficiency)	Moderate	No	No	BMT is not the standard treatment for this IEI
6	Uncertain (Unclassified)	Normal	No	No	BMT is not the standard treatment for this IEI
7	*PIK3CD* GOF Heterozygous [AD]	Mild	Yes	No	Received BMT as indicated
8	*IL2RG *Hemizygous [XL]	Severe	Yes	Yes	Received gene therapy instead of BMT
9	Uncertain (Unclassified)	Severe	Yes	No	Received BMT as indicated
10	Uncertain (Unclassified)	Normal	No	No	BMT is not the standard treatment for this IEI
11	Uncertain (Predominantly antibody deficiency)	Moderate	No	No	BMT is not the standard treatment for this IEI
12	Uncertain (Predominantly antibody deficiency)	Mild	No	Yes	Accelerated decision for BMT but did not get due to nutritional status
13	*ZAP70 *Compound heterozygous [AR]	Moderate	Yes	No	Received BMT as indicated
14	*RAG1 *Homozygous [AR]	Severe	Yes	Yes	Conditioning regimen was modified
15	Uncertain (Predominantly antibody deficiency)	Mild	No	No	BMT is not the standard treatment for this IEI
16	*GTF3A *Compound Heterozygous [AR]	Severe	Yes	No	Received BMT as indicated
17	*RAG1 *Homozygous [AR]	Normal	Yes	No	Accelerated decision for BMT
18	STAT1 *GOF *Heterozygous [AD]	Moderate	No	Yes	Conditioning regimen was modified (decreased Busulfan dose)
19	*RAG1 *Compound heterozygous [AR]	Severe	Yes	No	Received BMT as indicated
20	*PIK3R1 *Heterozygous [AD]	Severe	No	No	BMT is not the standard treatment for this IEI
21	Uncertain (Unclassified)	Normal	No	No	BMT is not the standard treatment for this IEI
22	Uncertain (Unclassified)	Mild	Yes	No	Received BMT as indicated
23	*RAG1 *Compound heterozygous [AR]	Severe	Yes	No	Received BMT as indicated
24	*RAG1 *Homozygous [AR] *CFTR *Heterozygous [AR]	Severe	Yes	Yes	Conditioning regimen was modified
25	*IL2RG *Hemizygous [XL]	Severe	Yes	No	Received gene therapy as indicated
26	STAT1 GOF Heterozygous [AD]	Severe	Yes	No	Received BMT as indicated
27	*RAG1 *Compound Heterozygous [AR]	Normal	Yes	No	Received BMT as indicated
28	STAT1 GOF Heterozygous [AD]	Severe	Yes	No	Received BMT as indicated
29	Uncertain (Predominantly antibody deficiency)	Mild	No	No	BMT is not the standard treatment for this IEI
30	*RAG1 *Compound Heterozygous [AR]	Severe	Yes	No	Received BMT as indicated
31	*RAG1 *Compound Heterozygous [AR]	Severe	Yes	No	Received BMT as indicated
32	*RAG1 *Compound Heterozygous [AR]	Mild	Yes	No	Received BMT as indicated
33	*IKZF3 *Heterozygous [AD]	Moderate	No	No	BMT is not the standard treatment for this IEI
34	Uncertain (Unclassified)	No pulmonary function test done	No	No	BMT is not the standard treatment for this IEI
35	Uncertain (Predominantly antibody deficiency)	Moderate	No	No	BMT is not the standard treatment for this IEI

*AD*: autosomal dominant; *AR*: autosomal recessive; *BMT*: bone marrow transplant; *GOF*: gain of function; *HCT-CI*: hematopoietic cell treatment-comorbidity index; *IEI*: inborn error of immunity; *XL*: X-linked

## Discussion

One in four patients in this genetically characterized, prospectively followed international IEI cohort, spanning 17 countries across six continents, had CT-confirmed bronchiectasis, representing more than 30 distinct genetic diagnoses. Older age at first chest CT, combined cellular and humoral immunodeficiency, predominantly antibody deficiencies, and diseases of immune dysregulation independently predicted bronchiectasis overall, with female sex and predominantly antibody deficiencies emerging as the dominant predictors in children and adults respectively. Pulmonary nodules, ground glass opacification, and lymphadenopathy were the most common co-existing CT findings. Bronchiectasis was associated with significantly greater impairment across every measured cardiopulmonary domain and fivefold excess mortality, and directly modified curative treatment strategy in 20% of eligible patients.

Published bronchiectasis prevalence in IEI ranges from 10% to 82%, reflecting disease heterogeneity, phenotypic selection, and variation in radiological ascertainment [24]. Our estimate of 27% derives from a quaternary specialist center population in whom chest CT was clinically indicated and likely represents an upper bound relative to unselected IEI populations. Nonetheless, the burden is substantial and consistent across diagnostic classes.

The independent association of older age with bronchiectasis is biologically plausible since repeated infections and chronic inflammation accumulate over time, progressively overwhelming the lung’s capacity for repair. Prior studies have compared median ages in patients with and without bronchiectasis but have not modelled age robustly in a multivariable framework adjusted simultaneously for IUIS class, sex and race [25]. Predominantly antibody deficiencies and diseases of immune dysregulation as predictors aligns with established pathobiology because impaired opsonization facilitates recurrent lower respiratory tract infections, while dysregulated inflammation drives airway damage independent of infectious burden [26–33]. The finding that female children with IEI carry higher bronchiectasis risk than male counterparts, independent of race and IUIS class, is consistent with emerging evidence of sex differences in bronchiectasis biology and warrants prospective investigation [34–36]. Notably, one patient with *RAG1* deficiency was also identified as a CF carrier, a combination that may synergistically increase bronchiectasis risk [37], though the clinical implications of this interaction require further study. Larger, globally representative IEI cohorts are needed to establish the true prevalence and full spectrum of bronchiectasis predictors across the life course.

The radiological triad of nodules, ground glass opacification, and lymphadenopathy, observed frequently in this cohort and documented previously in adults and children is suggestive of GLILD [38, 39], a recognized complication of CVID and other IEI [15]. Its identification should prompt immunological evaluation when no alternative explanation is apparent. Advanced bronchoscopic platforms including endobronchial ultrasound and CT navigational cryobiopsy offer opportunities to obtain tissue samples, characterize nodal and parenchymal pathology, exclude malignancy, and elucidate the underlying immunopathology to guide precise treatment in immunocompromised patients [40–42].

The cardiopulmonary complication data presented here are the most comprehensive in IEI to date [32]. Mixed obstructive-restrictive spirometry was confined exclusively to patients with bronchiectasis; abnormal DLCO was present in 60% vs. 17%; and echocardiographic pulmonary hypertension in 24% vs. 7%. These data support protocolized surveillance incorporating lung function testing and echocardiography across IEI population, not merely symptomatic assessment [53, 39]. The pulmonary-specific HCT-CI showed moderate discordance with the ATS/ERS severity classifications in a subset of patients [17, 22]. This exposes a clinically relevant limitation of the HCT-CI: it does not incorporate global race-neutral reference equations and Z-scores, nor account for lung restriction and hyperinflation, introducing misclassification risk with direct consequences for transplantation eligibility. Mortality was significantly greater among patients with bronchiectasis (15% vs. 3%), consistent with prior reports [43].

Only one third of patients received high-dose immunoglobulin replacement, despite evidence that patients with CVID and bronchiectasis require substantially higher doses to achieve equivalent IgG levels, likely reflecting increased catabolism in the setting of chronic lung disease [44, 45]. Published recommendations support an initial IVIG dose of at least 0.6 g/kg/month for patients with bronchiectasis, titrated with breakthrough infections [44], and alternative routes of administration may achieve higher IgG levels. The ongoing NCT07048262 trial will determine the dose range, efficacy and safety of nebulized immunoglobulin for adults with non-CF bronchiectasis. Similarly, only half of patients were on long-term azithromycin, despite evidence of exacerbation reduction in primary antibody deficiency [46], and endorsement across international non-CF bronchiectasis guidelines [1, 14, 47]. Both represent modifiable gaps in care.

Allogenic BMT has been shown to stabilize bronchiectasis [48], and normalize DLCO a decade post-transplant [49], supporting accelerated referral in selected patients with progressive lung disease [49, 50]. Conversely, established bronchiectasis may increase post-transplant pulmonary complication risk and, in severe cases, constitutes a contraindication to BMT [50], as in one patient in this cohort. Bronchiectasis modified curative treatment strategy in 20% of eligible patients, through contraindication, acceleration, choice of modality, or conditioning modification, underscoring the importance of systematic pre-transplant pulmonary assessment as a standard component of IEI evaluation. These findings collectively argue for protocolized bronchiectasis surveillance across IEI diagnostic classes, irrespective of symptomatic status, given that early detection is particularly consequential in children, in whom timely intervention may halt or reverse disease progression before irreversible structural injury narrow therapeutic options. As the therapeutic landscape evolves with emerging agents including Cathepsin C inhibitors [51] and autologous P63^+^ progenitor cell transplantation [52], patients with IEI and bronchiectasis should be prioritized in clinical trials.

Several limitations merit acknowledgement. The cohort was predominantly white, with over-representation of SCID, particularly *RAG* defects, and no patients with phagocyte defects, reflecting the research interests of the group and introducing referral bias. The 59% genetic diagnosis rate should be interpreted in the context of the quaternary immunological centre with preferential access to comprehensive genomic platforms, which likely inflates molecular characterization rates relative to broader clinical practice. The requirement for chest CT as an inclusion criterion may inflate bronchiectasis prevalence estimates relative to the broader IEI population. CT images were not centrally reviewed. The unclassified subgroup (33%), remains incompletely phenotyped owing to variable availability of granular immunological data. The cross-sectional design precludes causal inference, residual confounding cannot be excluded, and the study may have been underpowered to detect additional bronchiectasis predictors, particularly in subgroup analyses. Smoking exposure, occupational exposures, and concomitant pulmonary disorders were not systematically collected, precluding their inclusion as covariates in the multivariable models. The absence of prospectively collected data on symptom onset, first healthcare contact, and treatment initiation precluded analysis of diagnostic and treatment delay as potential risk factors for bronchiectasis, an important objective for future prospective studies. Several patients without bronchiectasis lacked cardiopulmonary outcome data, reflecting real-world practice. Immunoglobulin therapy could not be disaggregated by indication or dosing strategy, a distinction with direct clinical relevance given differing therapeutic thresholds across indications. Notwithstanding these limitations, this is one of the largest genotype-phenotype-imaging analyses in IEI, providing robust multivariable estimates of bronchiectasis predictors and the first explicitly evaluation of its influence on curative treatment decision-making.

## Conclusion

Bronchiectasis is a prevalent and clinically significant complication in children and adults with IEI within this NIH cohort. Age at first chest computed tomography and specific IUIS classes: immunodeficiencies affecting cellular and humoral immunity, predominantly antibody deficiencies and diseases of immune dysregulation are positive predictors of bronchiectasis in IEI patients. Cardiopulmonary complications and deaths are significantly greater in patients with bronchiectasis. High quality evidence for interventions encompassing the full clinical spectrum of therapeutic intent, from primary prevention in at-risk patients, to halting or slowing progression in established disease and, in young children whose airways retain greater plasticity, the potential reversal of existing structural changes, is urgently needed in this susceptible population.

## Data Availability

Data is provided within the manuscript or supplementary information files.

## Declarations

This study was approved by the National Institutes of Health Institutional Review Board. All study participants, or their parents provided written informed consent and age-appropriate assent according to the Declaration of Helsinki.
